# Is Better Diet Quality Linked to Better Competitive Functioning? An Integrative Multi-Analytic Study of Sleep Difficulty and Subjective Vitality Among Chinese Collegiate Athletes

**DOI:** 10.3390/nu18203385

**Published:** 2026-10-15

**Authors:** Tian Yin, Jiahao Wang, Tiande Pan, Pengbo Wang, Yanfeng Peng, Zeng Gao

**Affiliations:** 1 Department of Physical Education, Xiangtan University, Xiangtan 411105, China; shanliangdenanziha@163.com; 2 School of Physical Education, Yunnan Minzu University, Kunming 650504, China; 24207037980007@ymu.edu.cn; 3 School of Physical Education, China University of Geosciences (Wuhan), Wuhan 430074, China; wangjiahao@cug.edu.cn; 4 Institute of Strategic Development, Beijing Sport University, Beijing 100084, China; 5 Department of Postdoctoral, School of Marxism, Xiangtan University, Xiangtan 411105, China

**Keywords:** diet quality, sleep difficulty, subjective vitality, self-rated physical fitness, competitive psychological readiness, mental toughness, collegiate athletes

## Abstract

**Background:** Diet quality may be associated with recovery-related experiences and self-reported physical and psychological functioning in athletes. However, the roles of sleep difficulty and subjective vitality in these associations, together with their item-level network structure and predictive relevance, have not been examined within an integrated analytical framework. This study investigated the associations of diet quality with self-rated physical fitness and competitive psychological readiness among collegiate athletes and examined cross-sectional indirect associations involving sleep difficulty and subjective vitality. **Methods:** A cross-sectional survey was conducted among 1641 collegiate athletes participating in provincial-level university sporting events in Yunnan and Guizhou, China. Assessments were completed on the athletes’ competition check-in day. All core variables were assessed using self-report instruments, including diet quality, sleep difficulty, subjective vitality, self-rated physical fitness, and competitive psychological readiness, with the latter operationalized using mental toughness. Cross-sectional indirect associations were estimated using PROCESS Model 6. Regularized partial-correlation networks and bridge centrality analyses were used to characterize item-level conditional associations. Ridge regression and XGBoost were further applied to evaluate out-of-sample predictive performance, and SHAP values were used to quantify the relative predictive contribution of variables within the XGBoost models. **Results:** Higher diet quality was significantly associated with fewer sleep difficulties and with higher subjective vitality, self-rated physical fitness, and competitive psychological readiness. Significant indirect associations were observed involving sleep difficulty alone, subjective vitality alone, and the ordered sleep difficulty–subjective vitality term in both outcome models; the subjective-vitality-only estimate was the largest. Network analysis indicated that sleep-difficulty nodes were predominantly characterized by negative bridging connections, whereas subjective-vitality nodes showed primarily positive bridging connections across communities. In the predictive analyses, Ridge regression achieved holdout *R*^2^ values of 0.415 for self-rated physical fitness and 0.341 for competitive psychological readiness. In exploratory XGBoost analyses, subjective vitality showed the highest relative SHAP importance in both models. **Conclusions:** Diet quality was associated with self-rated physical fitness and competitive psychological readiness, operationalized using mental toughness, with additional indirect associations involving sleep difficulty and subjective vitality. These findings provide a conceptual basis for considering nutrition, sleep, and daytime vitality within an integrated athlete-support framework. Their temporal ordering and the effects of modifying these factors require longitudinal and experimental testing.

## 1. Introduction

### 1.1. Competitive Functioning as a Conceptual Framework

Athletes’ functioning during training and competition reflects the interaction of physical capacities, psychological resources, health status, training load, and recovery within a specific sporting context [1,2,3]. Athlete health–performance models therefore treat health management, training decisions, and performance as components of an integrated system [1]. Consistent with this view, recovery–performance theory emphasizes the balance between competitive demands and the physiological and psychological resources available for recovery [2], while psychological status is integral to training engagement, coping with competitive stress, and adaptation to sport-related demands [3].

Against this background, competitive functioning (CF) is used in the present study as an organizing framework for considering physical and psychological resources relevant to training and competition. CF was not measured as a single composite construct and should not be equated with competition results, objective athletic performance, or comprehensive readiness for competition. The empirical analyses focused on two self-reported indicators within this framework: physical fitness (PF) and competitive psychological readiness (CPR).

More specifically, PF captures athletes’ subjective appraisal of their overall fitness, cardiorespiratory fitness, muscular strength, speed and agility, and flexibility [4,5]. These perceived capacities are relevant to how athletes evaluate their ability to meet the demands of training and competition and are closely linked to training adaptation and the execution of sport-related tasks [6]. Within the psychological domain, CPR was operationalized using mental toughness, whose dimensions of confidence, constancy, and control reflect athletes’ capacity to remain engaged, consistent, and in control under competitive pressure [7,8]. PF and CPR thus represent complementary self-reported physical and psychological resources within the broader CF framework. In the present study, PF therefore denotes perceived fitness, whereas CPR captures the aspect of competitive psychological readiness indexed by mental toughness.

Collegiate athletes are a particularly relevant population in which to examine CF because they must coordinate academic responsibilities with systematic training and competition. Within the dual-career framework, sustained demands across sport and education can constrain time and recovery opportunities and contribute to fatigue and psychological strain [9]. The university years also involve increasing responsibility for everyday health-related routines. Identifying modifiable factors associated with physical and psychological resources may therefore clarify how collegiate athletes function under sustained dual-career demands.

### 1.2. Diet Quality, Self-Rated Physical Fitness, and Competitive Psychological Readiness

Diet quality (DQ) reflects the extent to which an individual’s overall dietary pattern meets health and nutritional requirements. Rather than representing the intake of a single food or nutrient, DQ is a multidimensional construct encompassing food variety, nutrient intake, dietary balance, and consistency with established health recommendations [10]. It is commonly evaluated using composite indices that characterize whether habitual dietary intake is nutritionally adequate and health-promoting while limiting components potentially detrimental to health [10,11]. In athletes, DQ is particularly relevant to nutritional support for training adaptation, recovery, and long-term health [12]. In the present study, DQ was operationalized using a brief self-report screener of selected health-promoting and less healthful habitual dietary behaviors, rather than a quantitative assessment of food or nutrient intake, nutrient adequacy, or energy availability.

DQ may be associated with both self-rated PF and CPR, with the latter operationalized using mental toughness. From a physical perspective, an adequate and well-structured diet provides the nutritional foundation for training adaptation, muscle repair, immune maintenance, energy provision, and post-exercise recovery, and may therefore be associated with athletes’ perceived physical functioning [12]. Contemporary sport nutrition frameworks likewise position dietary intake as an important link between habitual eating behavior, recovery, training adaptation, and preparation for competition [13]. At a more specific physiological level, field evidence from female ultramarathon runners showed that exercise-related acid–base and electrolyte responses varied across performance groups during prolonged endurance exercise, illustrating the broader relevance of fluid–electrolyte regulation to athletic functioning [14]. From a psychological perspective, DQ may also be associated with the resources required to sustain confidence, concentration, and engagement through its relationships with perceived fatigue, recovery experiences, and day-to-day energy. Previous reviews indicate that athletes’ dietary intake does not consistently meet sport nutrition recommendations, while limited nutrition knowledge and access to professional support may further compromise DQ [15]. Examining the associations of DQ with PF and CPR may therefore clarify how dietary behavior relates to perceived physical functioning and the psychological resources captured by mental toughness.

This question is especially relevant to collegiate athletes, whose dietary choices are shaped by class and training schedules, financial constraints, and campus food environments [9,16]. Compared with professional or elite athletes, they often have less consistent access to systematic nutrition support, leaving dietary behavior more dependent on everyday contexts and established habits [17]. Examining its associations with PF and CPR may therefore inform future research on nutrition and functioning in this dual-career population.

### 1.3. Indirect Association Involving Sleep Difficulty

Sleep difficulty (SD) represents an important dimension of impaired sleep health, encompassing difficulties with sleep initiation and maintenance, reduced sleep quality, and associated daytime functional impairment [18,19]. In athletes, SD may reflect not only disrupted nocturnal sleep but also incomplete physiological, cognitive, and psychological recovery following training, making it a relevant indicator of compromised recovery status.

Beyond its implications for recovery, insufficient or poor-quality sleep has been associated with suboptimal physical adaptation, reduced cognitive functioning, and poorer athletic performance [2,18,19]. From a sport-recovery perspective, the functional consequences of training load depend partly on the adequacy of physiological and psychological recovery; when recovery is insufficient, fatigue may accumulate and constrain subsequent adaptation and performance [20,21]. Experimental evidence further illustrates the interdependence of sleep, recovery, and performance. In a randomized crossover trial involving physically active men, short-term creatine loading was associated with better subjective sleep quality, cognitive performance, and high-intensity exercise output and with lower muscle-soreness ratings, although objective sleep metrics and most recovery indices remained unchanged [22]. Within this framework, SD may indicate incomplete restoration of functional resources and may therefore be associated with both self-rated PF and CPR [23,24]. Dietary behavior may also be related to SD. Less favorable dietary patterns and specific dietary practices have been associated with poorer perceived recovery and sleep status [25,26]. Among 234 endurance athletes, greater consumption of caffeinated beverages was associated with poorer sleep quality, whereas higher whole-grain intake was associated with fewer sleep-related problems [27]. Complementing this observational evidence, a randomized crossover study in trained males examined nocturnal melatonin and next-morning caffeine within an integrated sleep–performance framework. Although sleep parameters did not differ across conditions, caffeine-containing conditions improved high-intensity exercise performance, and the combined regimen was associated with favorable changes in selected markers of muscle damage and inflammation [28]. Together, these findings provide a theoretical and empirical basis for examining indirect associations involving DQ, SD, and the two study outcomes.

Collegiate athletes may be particularly vulnerable to SD because academic and sporting schedules can disrupt opportunities for regular sleep [29]. Approximately one quarter of student-athletes report clinically meaningful sleep problems [30], underscoring the value of examining SD within the associations of DQ with PF and CPR.

### 1.4. Indirect Association Involving Subjective Vitality

Subjective vitality (SV) refers to an individual’s subjective sense of energy, aliveness, and vigor, reflecting the perceived availability of physical and psychological energy rather than objective physical capacity or transient positive affect [31]. In competitive sport settings, SV reflects the extent to which athletes feel sufficiently energized to remain actively engaged in training and competition and may therefore serve as an important psychological indicator of their current functional state [32].

DQ may covary with SV through day-to-day experiences of energy, fatigue, and recovery [33]. Among 73 college students, healthier dietary patterns were associated with higher SV, which showed a significant indirect association between DQ and subjective well-being [34]. Although obtained outside sport, this finding provides a basis for examining whether DQ and perceived energy are similarly related in athletes facing greater training demands.

SV may also relate to both self-rated PF and CPR. Self-determination theory positions vitality as an indicator of effective self-regulation and positive functioning that supports active, goal-directed engagement [35,36], and evidence from athletes links SV with performance [37]. Within the collegiate dual-career context, SV may be particularly responsive to dietary conditions and cumulative demands. Examining SV as a cross-sectional indirect correlate may therefore clarify how DQ, perceived energy, and the two outcomes are statistically connected, without implying temporal or causal ordering.

### 1.5. Theoretically Specified Ordering of Sleep Difficulty and Subjective Vitality

SD and SV represent two conceptually distinct levels of the recovery–resource process. SD reflects disruption to nocturnal recovery, whereas SV captures the physical and psychological energy perceived as available and mobilizable during waking hours. According to conservation of resources theory, restorative activities such as sleep help replenish resources depleted through everyday demands. Accordingly, impaired recovery may constrain resource restoration and reduce the energy available for subsequent goal-directed activities [38]. This distinction positions SD at the recovery-process level and SV at the perceived resource-availability level, providing a theoretical basis for specifying SD before SV. Previous research has demonstrated significant associations between sleep quality and SV, with sleep efficiency and sleep-related daytime dysfunction emerging as particularly relevant components [39]. Two diary studies by Rivkin et al. further showed that prior-night sleep duration was associated with next-morning SV and that SV exhibited a significant indirect association between sleep duration and subsequent work engagement [40]. Although this evidence was derived primarily from general adult populations rather than athletes, it supports the prespecified ordering used in the statistical model. However, because this ordering was imposed a priori on cross-sectional data, it does not establish that SD temporally precedes SV; reciprocal or alternative orderings remain plausible.

This reasoning supports examining a theoretically ordered cross-sectional indirect-association model involving DQ, SD, SV, and the two outcomes. Specifically, the present model evaluates whether the observed covariance pattern is statistically consistent with the theory-specified ordering of DQ, SD, SV, and the outcomes; it does not test temporal succession or causal transmission among these variables.

### 1.6. The Present Study

Taken together, previous research provides separate evidence for associations among DQ, SD, SV, PF, and CPR, yet their specific and theoretically ordered indirect associations have rarely been examined simultaneously in collegiate athletes. Existing evidence has also largely relied on correlations or regression analyses based on scale-level total scores, limiting the ability to identify specific items that may serve as cross-construct connections within the broader system. In addition, statistical association should not be equated with out-of-sample prediction, as explanation and prediction represent related but distinct analytical objectives [41,42].

To address these gaps, PROCESS Model 6 was used to estimate cross-sectional indirect associations. Regularized partial-correlation networks characterized item-level conditional associations and bridge nodes connecting construct communities [43,44,45]. Ridge regression and XGBoost then evaluated out-of-sample prediction of PF and CPR, with SHAP values describing model-specific predictor contributions within XGBoost [46,47,48]. These approaches addressed complementary questions concerning construct-level associations, item-level connectivity, and predictive performance.

**Figure 1:**
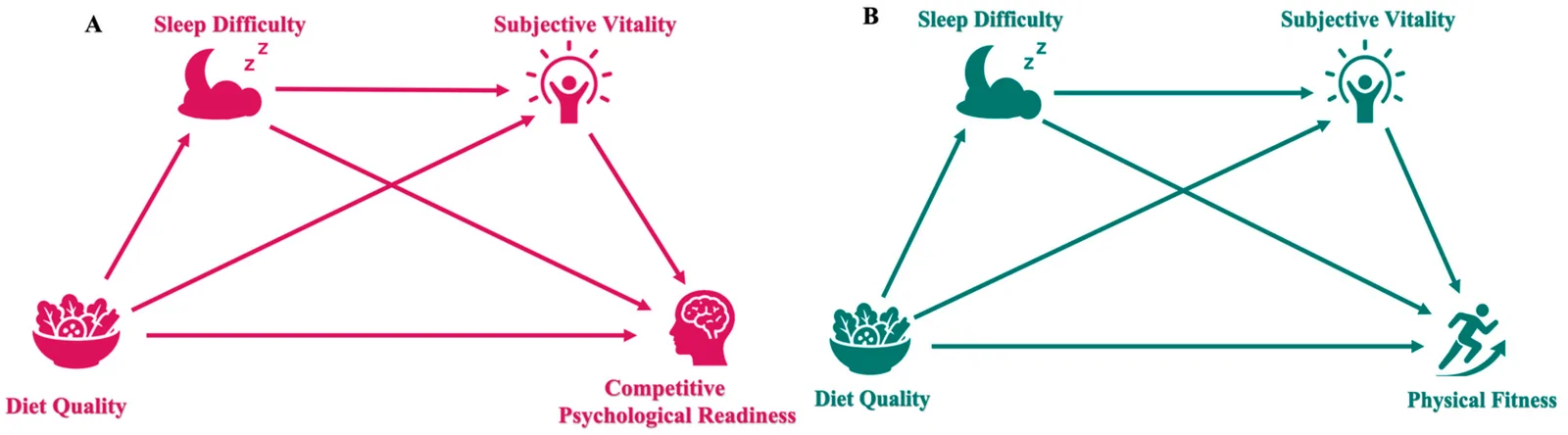
Hypothesized indirect-association models. (**A**) Model with competitive psychological readiness as the outcome; (**B**) model with self-rated physical fitness as the outcome.

The study included Chinese collegiate athletes participating in provincial-level sporting events and examined PF and CPR as two related self-reported outcomes within the broader CF framework. Neither outcome was treated as a direct measure of objective physical performance or comprehensive readiness for competition. As illustrated in Figure 1, the following hypotheses were proposed:

**H1a.**  *DQ is positively associated with PF*.

**H1b.**  *DQ is positively associated with CPR*.

**H2a.**  *An indirect association involving SD is expected between DQ and PF*.

**H2b.**  *An indirect association involving SD is expected between DQ and CPR*.

**H3a.**  *An indirect association involving SV is expected between DQ and PF*.

**H3b.**  *An indirect association involving SV is expected between DQ and CPR*.

**H4a.**  *An ordered indirect association involving SD and SV is expected between DQ and PF*.

**H4b.**  *An ordered indirect association involving SD and SV is expected between DQ and CPR*.

## 2. Methods

### 2.1. Participants

A convenience sampling approach was used to recruit collegiate athletes during provincial-level university sporting events held in Yunnan and Guizhou, China, between March and June 2026. Participants were university students engaged in systematic sport-specific training who represented their institutions in these provincial-level university competitions. All questionnaires were administered on the competition check-in day at the athletes’ hotels or designated registration sites after they had arrived at the event location and before their scheduled competitions. This time point was selected because it provided a common and logistically feasible assessment window across sports and event dates, allowing data to be collected within a broadly comparable competition-related context.

Participants were recruited in person by trained research staff at the athletes’ hotels or designated registration sites. Eligible participants accessed the electronic questionnaire through a survey link or QR code generated using the Wenjuanxing platform and completed it independently on their mobile phones.

Before data collection, participants were informed of the main purpose of the study, the anonymous nature of participation, data confidentiality procedures, and the intended use of the collected information. Participation was entirely voluntary, and participants were informed that they could withdraw at any stage without penalty. An informed-consent statement was presented on the first page of the electronic questionnaire, and only records accompanied by explicit consent were eligible for inclusion in the analytic sample. All research personnel involved in questionnaire administration and collection received standardized training covering participant instructions, on-site procedures, response to participant queries, and data-quality control requirements.

The inclusion criteria were as follows: (1) current enrollment as a university student; (2) athlete status and participation in the provincial sporting events held in Yunnan or Guizhou; (3) sufficient reading comprehension to complete the electronic questionnaire independently; and (4) provision of informed consent and voluntary participation. The exclusion criteria were: (1) failure to provide explicit informed consent; (2) clearly incomplete questionnaire responses; (3) missing data on the core study variables; (4) an implausibly short completion time or obvious inconsistencies across responses; and (5) failure to meet the predefined characteristics of the target population.

An a priori power analysis for the primary adjusted regression model was conducted using G*Power 3.1.9.7 (linear multiple regression: fixed model, *R*^2^ deviation from zero). The calculation included 11 predictor terms because the three-category sport-type variable was represented by two dummy variables. Assuming a small effect size (*f*^2^ = 0.02), *α* = 0.05, and 90% power, the minimum required sample size was 1070. Allowing for up to 20% unusable responses yielded a minimum recruitment target of 1338. Because convenience sampling was used, all eligible athletes accessible at the selected events during the prespecified recruitment period were invited. The final analytic sample of 1641 exceeded this requirement; the stability of the complementary network and predictive analyses was further examined using bootstrap and repeated-resampling procedures.

A total of 1827 survey records were received. After screening, 186 records were excluded according to the prespecified criteria, with each assigned a single primary reason for exclusion: 23 lacked explicit informed consent, 32 were clearly incomplete, 84 had missing data on at least one core study variable, 25 showed implausibly short completion times or internally inconsistent response patterns, and 22 did not meet the target-population eligibility criteria. The final analytic sample comprised 1641 participants with complete data for all core variables and prespecified covariates, yielding a valid-response rate of 89.82%. All analyses were based on this common complete-case sample, and no missing-data imputation was performed. The sample comprised 1100 men (67.03%) and 541 women (32.97%), with a mean age of 20.53 ± 1.46 years. The study was conducted in accordance with the principles of voluntary participation, anonymity, and data confidentiality and was approved by the Science and Technology Ethics Committee of Yunnan Minzu University (approval no. 2026021).

### 2.2. Measurement Instruments

All instruments were administered in Simplified Chinese. Published Chinese versions were used for the ASSQ and IFIS. Because validated Chinese versions were unavailable for the exact REAP-S, six-item state SVS, and original 14-item SMTQ forms used in this study, these three measures underwent a standardized study-specific translation and cultural-adaptation procedure. Two bilingual researchers independently translated the items and reconciled discrepancies by consensus, after which an independent bilingual researcher back-translated the reconciled versions. The research team compared the back-translations with the original instruments and reviewed the final Chinese wording for conceptual equivalence, cultural appropriateness, and clarity before data collection.

#### 2.2.1. Diet Quality

Diet quality was assessed with the 13-item Rapid Eating Assessment for Participants–Shortened Version (REAP-S) [49]. All scored items refer to eating practices during an average week and are worded in the direction of less favorable dietary behavior. Items concerning whole grains, fruit, vegetables, and dairy ask whether intake is below two servings per day. The study-specific Simplified Chinese adaptation retained the original item direction, response format, and scoring, with only selected food examples adapted to the Chinese dietary context. Responses were scored as 1 (“usually/often”), 2 (“sometimes”), and 3 (“rarely/never” or “does not apply to me”); no items were reverse-scored. Scores were summed (range: 13–39), with higher scores indicating better diet quality. As a brief screener, the REAP-S characterizes habitual dietary behaviors but does not quantify dietary or nutrient intake, nutrient adequacy, energy availability, or physiological recovery processes.

Previous studies have demonstrated associations of REAP-S scores with the Healthy Eating Index and other diet-quality indicators [50]. A score of approximately 32—the mean reported for adults consuming a typical omnivorous diet—has been used as a descriptive benchmark [51]. Accordingly, the sample distribution was summarized using scores below (<32) and at or above (≥32) this benchmark, while DQ was retained as a continuous variable in all primary analyses.

In the present sample, the REAP-S demonstrated acceptable internal consistency, with a Cronbach’s *α* of 0.793 and a Kaiser–Meyer–Olkin (KMO) value of 0.898. Confirmatory factor analysis indicated an acceptable model fit: *χ*^2^/df = 4.121, RMSEA = 0.044, SRMR = 0.036, CFI = 0.935, TLI = 0.922, AVE = 0.530, and CR = 0.794.

#### 2.2.2. Sleep Difficulty

Sleep difficulty was assessed using the Athlete Sleep Screening Questionnaire (ASSQ) originally developed by Samuels et al. [52]. The administered Chinese Sleep Difficulty Score followed the item composition and scoring of the ASSQ-CHN, which was cross-culturally adapted and demonstrated acceptable measurement properties among Chinese elite athletes [53]. The Sleep Difficulty Score (SDS) was derived from ASSQ items 1, 3, 4, 5, and 6 and comprised five components covering sleep duration, subjective sleep quality, sleep-onset latency, sleep maintenance, and use of sleep medication. Total scores range from 0 to 17, with higher scores indicating greater sleep difficulty. Scores of 0–4, 5–7, 8–10, and 11–17 correspond to no apparent, mild, moderate, and severe sleep difficulty, respectively. The items referred to sleep patterns in the “recent past,” without specifying a fixed number of days.

Clinical validation research has demonstrated good agreement between the ASSQ and assessments made by sleep-medicine physicians, with a weighted κ of 0.84, sensitivity of 81%, and specificity of 93% [54]. In the present study, the sleep-difficulty component of the ASSQ showed acceptable internal consistency, with Cronbach’s *α* = 0.755 and KMO = 0.791. Confirmatory factor analysis further indicated satisfactory psychometric performance: *χ*^2^/df = 6.640, RMSEA = 0.059, SRMR = 0.027, CFI = 0.985, TLI = 0.969, AVE = 0.503, and CR = 0.758.

#### 2.2.3. Subjective Vitality

Subjective vitality was assessed using a study-specific Simplified Chinese adaptation of the six-item state Subjective Vitality Scale (SVS) [31,55]. Although a five-item Chinese version has demonstrated satisfactory psychometric properties [56], it does not correspond to the six-item state form used in the present study. The administered items referred to participants’ subjective energy and vitality during the preceding week. Each item was rated on a 7-point Likert scale ranging from 1 (“not at all true”) to 7 (“very true”).

In the present study, the SVS demonstrated good internal consistency, with Cronbach’s *α* = 0.840 and KMO = 0.877. Confirmatory factor analysis showed an acceptable model fit: *χ*^2^/df = 8.387, RMSEA = 0.067, SRMR = 0.025, CFI = 0.979, TLI = 0.966, AVE = 0.568, and CR = 0.840.

#### 2.2.4. Mental Toughness as an Indicator of Competitive Psychological Readiness

Competitive psychological readiness was operationalized using a study-specific Simplified Chinese adaptation of the original 14-item Sports Mental Toughness Questionnaire (SMTQ) developed by Sheard et al. [8]. The SMTQ assesses general tendencies in confidence, constancy, and control across training and competitive settings, without specifying a fixed retrospective interval. These dimensions represent psychological resources relevant to maintaining engagement and perceived control under competitive demands, providing the rationale for using the SMTQ total score as a focused indicator of CPR. However, mental toughness is not synonymous with comprehensive competitive psychological readiness and does not capture all state-related, affective, tactical, or situational aspects of readiness for competition. Accordingly, CPR in the present study refers specifically to a mental-toughness-based operational indicator.

The SMTQ consists of 14 items covering three dimensions: Confidence, Constancy, and Control. Items are rated on a 4-point Likert scale ranging from 1 (“not at all true”) to 4 (“very true”). Items 2, 4, 7, 8, 9, and 10 are reverse scored. Item scores are summed to produce a total score ranging from 14 to 56, with higher scores indicating greater mental toughness, which served as the operational indicator of CPR in the present study.

In the present sample, the SMTQ demonstrated good internal consistency, with Cronbach’s *α* = 0.901 and KMO = 0.958. Confirmatory factor analysis indicated a satisfactory model fit: *χ*^2^/df = 4.776, RMSEA = 0.048, SRMR = 0.032, CFI = 0.962, TLI = 0.956, AVE = 0.593, and CR = 0.899.

#### 2.2.5. Self-Rated Physical Fitness

Self-rated physical fitness was assessed using the Simplified Chinese version of the International Fitness Scale (IFIS-C) [57,58]. The IFIS-C was developed through independent forward translations, reconciliation, and back-translation and has demonstrated moderate test–retest reliability among Chinese children and adolescents [58]. Because this evidence was obtained in a younger population, the reliability and internal structure of the scale were additionally examined in the present sample. It covers overall physical fitness, cardiorespiratory fitness, muscular strength, speed and agility, and flexibility. The items elicited a global self-assessment of current physical fitness without specifying a fixed retrospective interval. The scale contains five items rated on a 5-point Likert scale ranging from 1 (“very poor”) to 5 (“very good”). Item scores are summed to generate a total score ranging from 5 to 25, with higher scores indicating better self-rated physical fitness.

In the present study, the IFIS showed acceptable internal consistency, with Cronbach’s *α* = 0.780 and KMO = 0.818. Confirmatory factor analysis indicated a satisfactory model fit: *χ*^2^/df = 6.153, RMSEA = 0.056, SRMR = 0.022, CFI = 0.987, TLI = 0.974, AVE = 0.523, and CR = 0.783.

### 2.3. Covariates

Covariates were selected from the demographic and habitual training characteristics recorded in the survey. Sex, age, body mass index (BMI), formal athlete classification, daily training duration, weekly training frequency, and sport type were included because they may be associated with DQ, SD, SV, PF, and CPR among collegiate athletes. Adjustment for these variables was intended to reduce confounding related to demographic background and habitual training exposure; situational, nutritional, psychological, and clinical factors not included in the survey remained potential sources of residual confounding.

### 2.4. Data Processing and Statistical Analysis

Data processing and statistical analyses were performed using SPSS version 26.0, R version 4.3.1, and Python 3.9. The distributions of continuous variables were examined using means, standard deviations, skewness, kurtosis, and Q–Q plots. Sex differences in continuous variables were assessed using Welch’s independent-samples *t* tests, with Hedges’ *g* reported as the standardized mean difference. Categorical variables were compared using Pearson’s *χ*^2^ tests, with Cramér’s *V* reported as the corresponding effect size. Internal consistency was evaluated using Cronbach’s α. Confirmatory factor analysis (CFA) was conducted using maximum likelihood estimation, with model fit evaluated using *χ*^2^/df, the root mean square error of approximation (RMSEA), standardized root mean square residual (SRMR), comparative fit index (CFI), Tucker–Lewis index (TLI), average variance extracted (AVE), and composite reliability (CR). Because all core variables were derived from the same source and assessed using self-report measures, an unmeasured latent method construct (ULMC) model was additionally employed to evaluate potential common method variance [59]. Bivariate associations among the principal study variables were examined using Pearson correlations.

Indirect associations were estimated using PROCESS Model 6, with PF and CPR analyzed separately. DQ was specified as the predictor, SD and SV as ordered intermediate variables, and PF or CPR as the outcome, with adjustment for sex, age, BMI, daily training duration, weekly training frequency, formal athlete classification, and sport type. The SD–SV ordering was prespecified and does not establish temporal precedence. Estimates and 95% confidence intervals (CIs) were obtained using 5000 bootstrap resamples [60]. Given the large sample size, statistical interpretation emphasized standardized coefficient magnitudes and 95% CIs alongside *p* values rather than relying on statistical significance alone.

Item-level network analyses were performed in R using the qgraph, bootnet, and networktools packages. All questionnaire items were treated as ordinal variables, and the correlation matrix was estimated using cor_auto, which automatically selects an appropriate correlation coefficient according to variable characteristics. Regularized partial-correlation networks were estimated using the Extended Bayesian Information Criterion graphical least absolute shrinkage and selection operator (EBICglasso), with the EBIC tuning parameter set to γ = 0.50. Communities were defined a priori according to scale membership. Bridge Strength and one-step Bridge Expected Influence were calculated to characterize cross-community connectivity.

The accuracy of estimated network edge weights was evaluated using 1000 nonparametric bootstrap resamples. The stability of the conventional centrality indices Strength and Expected Influence was assessed using case-dropping bootstrap procedures and quantified using the correlation stability coefficient (CS coefficient). Higher CS coefficients indicate that centrality rankings remain more consistent after progressively larger proportions of the sample are removed. Bridge Strength and one-step Bridge Expected Influence were used to describe the extent and direction of connections between nodes and other predefined communities. However, these bridge centrality indices were not separately evaluated using case-dropping bootstrap procedures. Their magnitudes and relative rankings were therefore interpreted descriptively rather than as node rankings supported by independent stability testing.

To complement the preceding association and network analyses, Ridge regression and XGBoost models were fitted separately for PF and CPR to evaluate how well DQ, SD, SV, and the demographic and training variables jointly predicted each outcome in held-out observations. With a modest set of potentially correlated predictors, Ridge regression served as the regularized linear benchmark [47]. XGBoost was included as a flexible nonlinear model to assess whether nonlinearities and interactions improved prediction beyond this benchmark [46]. Within each training set, the Ridge penalty was selected by generalized cross-validation across 80 logarithmically spaced values (10^−3^–10^4^). Both models were fitted to the same prespecified 80% training set and evaluated on the remaining 20% (seed 20260716) using RMSE, MAE, and *R*^2^; five-fold cross-validation was additionally performed within the training set. Sensitivity to sample allocation was assessed across 100 repeated 80/20 splits (seeds 20260716–20260815), with preprocessing and model fitting repeated within each training partition and test performance summarized by the mean, SD, and empirical 2.5th–97.5th percentiles. Given its exploratory role, XGBoost retained prespecified hyperparameters rather than undergoing nested tuning. SHAP values from the prespecified XGBoost model fitted to the original split were used to characterize model-specific predictor contributions [48].

## 3. Results

### 3.1. Assessment of Common Method Variance

Because all study variables were assessed using self-report measures obtained from the same source, an unmeasured latent method construct (ULMC) approach was used to evaluate potential common method variance. The baseline five-factor measurement model showed a good fit to the data (χ^2^/df = 2.449, RMSEA = 0.030, SRMR = 0.031, CFI = 0.943, TLI = 0.939). After inclusion of a common method factor, model fit improved (χ^2^/df = 1.916, RMSEA = 0.024, SRMR = 0.026, CFI = 0.966, TLI = 0.961), with the method factor accounting for an average of 2.1% of the variance. Although the proportion of variance attributable to the method factor was small, the improvement in model fit indicates that common method variance cannot be entirely ruled out. This statistical assessment does not eliminate recall bias, social-desirability bias, response-style effects, or other sources of bias arising from same-source self-report measurement. Associations among variables derived from the same self-report source should therefore be interpreted with appropriate caution [61].

### 3.2. Participant Characteristics and Sex Differences in the Main Study Variables

As shown in Table 1, substantial interindividual variation was observed in DQ, SD, SV, PF, and CPR among the 1641 collegiate athletes. All participants were engaged in systematic sport-specific training and represented their institutions in provincial competition, although 76.42% did not hold a formal national athlete classification. Overall, 33.46% of participants scored at or above the REAP-S descriptive reference value of 32, while 25.17% exhibited moderate or severe SD. Female athletes reported higher DQ and SD, whereas male athletes reported higher SV, self-rated PF, and CPR, with CPR operationalized using mental toughness (all *p* < 0.05). These differences were small (|Hedges’ *g*| = 0.13–0.25). Age, BMI, daily training duration, and weekly training frequency did not differ by sex and showed negligible effects (|*g*| ≤ 0.06); nor did academic grade, formal athlete classification, or sport type (Cramér’s *V* = 0.03–0.05). Although the descriptive DQ grouping and SD categories differed significantly by sex, the effects were small (Cramér’s *V* = 0.09 and 0.11, respectively).

### 3.3. Correlation Analysis

Pearson correlation analyses (Table 2) showed that DQ was significantly and negatively correlated with SD (*r* = −0.282, *p* < 0.001) and significantly and positively correlated with SV, PF, and CPR (*r* = 0.295–0.453, all *p* < 0.001). SD was significantly and negatively correlated with SV, PF, and CPR (*r* = −0.340 to −0.479, all *p* < 0.001). SV showed relatively strong positive correlations with both PF (*r* = 0.597) and CPR (*r* = 0.580). PF was also significantly and positively correlated with CPR (*r* = 0.556, *p* < 0.001).

### 3.4. Indirect-Association Analysis

As shown in Table 3, PROCESS Model 6 was used to estimate indirect associations involving SD and SV after adjustment for the prespecified covariates.

DQ was negatively associated with SD (*β* = −0.299, 95% CI [−0.345, −0.253], *p* < 0.001) and positively associated with SV (*β* = 0.354, 95% CI [0.313, 0.396], *p* < 0.001), whereas SD was negatively associated with SV (*β* = −0.370, 95% CI [−0.412, −0.329], *p* < 0.001).

**Figure 2:**
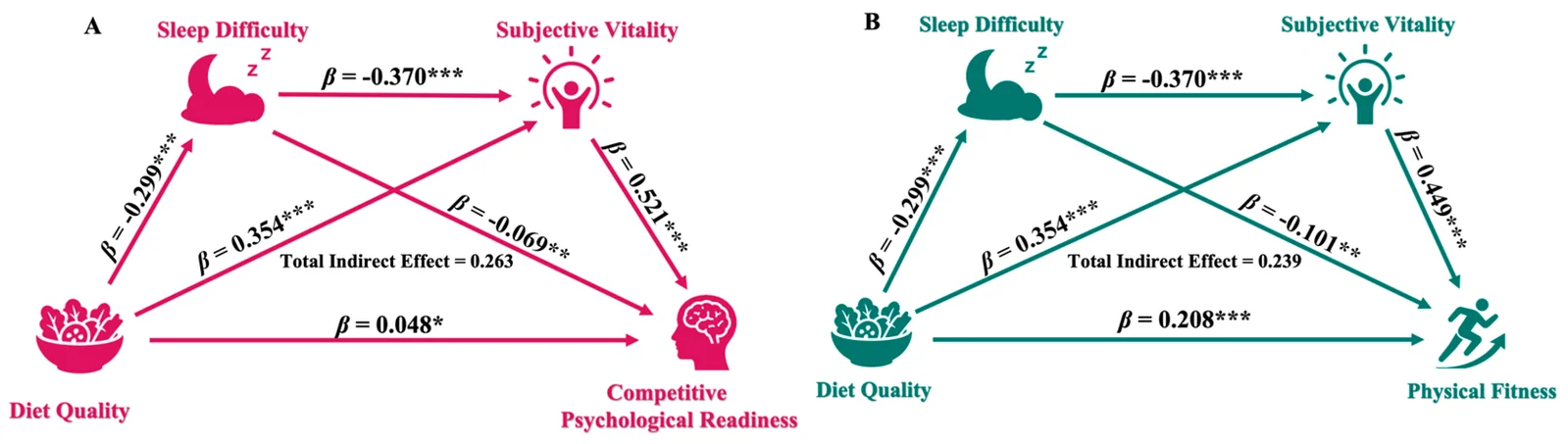
Standardized coefficients and total indirect effect. (**A**) Serial mediation model predicting competitive psychological readiness; (**B**) serial mediation model predicting self-perceived physical fitness. * *p* < 0.05, ** *p* < 0.01, *** *p* < 0.001.

In the self-rated PF model, DQ (*β* = 0.208, 95% CI [0.166, 0.250]), SD (*β* = −0.101, 95% CI [−0.143, −0.058]), and SV (*β* = 0.449, 95% CI [0.403, 0.494]) were associated with PF (all *p* < 0.001), with SV showing the largest standardized coefficient. In the CPR model, in which CPR was operationalized using mental toughness, the adjusted DQ–CPR association was positive but small (*β* = 0.048, 95% CI [0.003, 0.093], *p* = 0.038). SD showed a small negative association with CPR (*β* = −0.069, 95% CI [−0.114, −0.024], *p* = 0.003), whereas SV showed the largest positive coefficient (*β* = 0.521, 95% CI [0.473, 0.570], *p* < 0.001). The standardized path coefficients are presented in Figure 2.

As reported in Table 4, the total indirect-association estimate was 0.239 for PF (95% CI [0.212, 0.267]) and 0.263 for CPR (95% CI [0.233, 0.293]). For both outcomes, the estimates involving SD alone, SV alone, and the ordered SD–SV term had bootstrap CIs excluding zero. Pairwise comparisons showed that the SV-only estimate exceeded both the SD-only and ordered SD–SV estimates. These differences reflect model-based cross-sectional associations, not temporal or causal importance.

### 3.5. Network Analysis

#### 3.5.1. Item Coding and Network Estimation

To characterize item-level associations among DQ, SD, SV, PF, and CPR, separate regularized partial-correlation networks were constructed for PF and CPR. Network nodes were coded a priori according to their corresponding scales. DQ comprised breakfast regularity (DQ1), limited fast-food consumption (DQ2), adequate whole-grain intake (DQ3), adequate fruit intake (DQ4), adequate vegetable intake (DQ5), adequate dairy intake (DQ6), moderate meat intake (DQ7), limited processed-meat consumption (DQ8), limited fried-food consumption (DQ9), limited high-fat snack consumption (DQ10), limited added fat intake (DQ11), limited sweets consumption (DQ12), and limited sugar-sweetened beverage consumption (DQ13). SD comprised short sleep duration (SD1), dissatisfaction with sleep quality (SD2), sleep-onset latency (SD3), sleep-maintenance difficulty (SD4), and use of sleep medication (SD5). SV comprised aliveness (SV1), bursting energy (SV2), energetic state (SV3), daily anticipation (SV4), alertness (SV5), and vigor (SV6). In the PF network, PF was represented by five self-rated IFIS items: perceived overall fitness (PF1), perceived cardiorespiratory fitness (PF2), perceived muscular strength (PF3), perceived speed and agility (PF4), and perceived flexibility (PF5). In the CPR network, CPR was represented by 14 SMTQ items spanning confidence, constancy, and control: composure recovery (CPR1), performance assurance (CPR2), task persistence (CPR3), self-belief resilience (CPR4), ability confidence (CPR5), performance under pressure (CPR6), emotional control (CPR7), perseverance (CPR8), anxiety control (CPR9), attentional control (CPR10), perceived competitive advantage (CPR11), challenging goal setting (CPR12), viewing threats as opportunities (CPR13), and confident decision-making under pressure (CPR14).

The PF network consisted of four communities: DQ, SD, SV, and PF, whereas the CPR network comprised DQ, SD, SV, and CPR. Blue and red edges represented positive and negative conditional associations, respectively, after adjustment for all other nodes in the network. Edge width and color intensity reflected the absolute magnitude of the regularized partial correlations. Because the networks contained both positive and negative edges, Bridge Strength and one-step Bridge Expected Influence were reported simultaneously. Bridge Strength reflects the overall magnitude of a node’s connections with other communities, whereas Bridge Expected Influence retains edge direction and therefore captures the net positive or negative cross-community connectivity of each node.

#### 3.5.2. Main Network Structure

**Figure 3:**
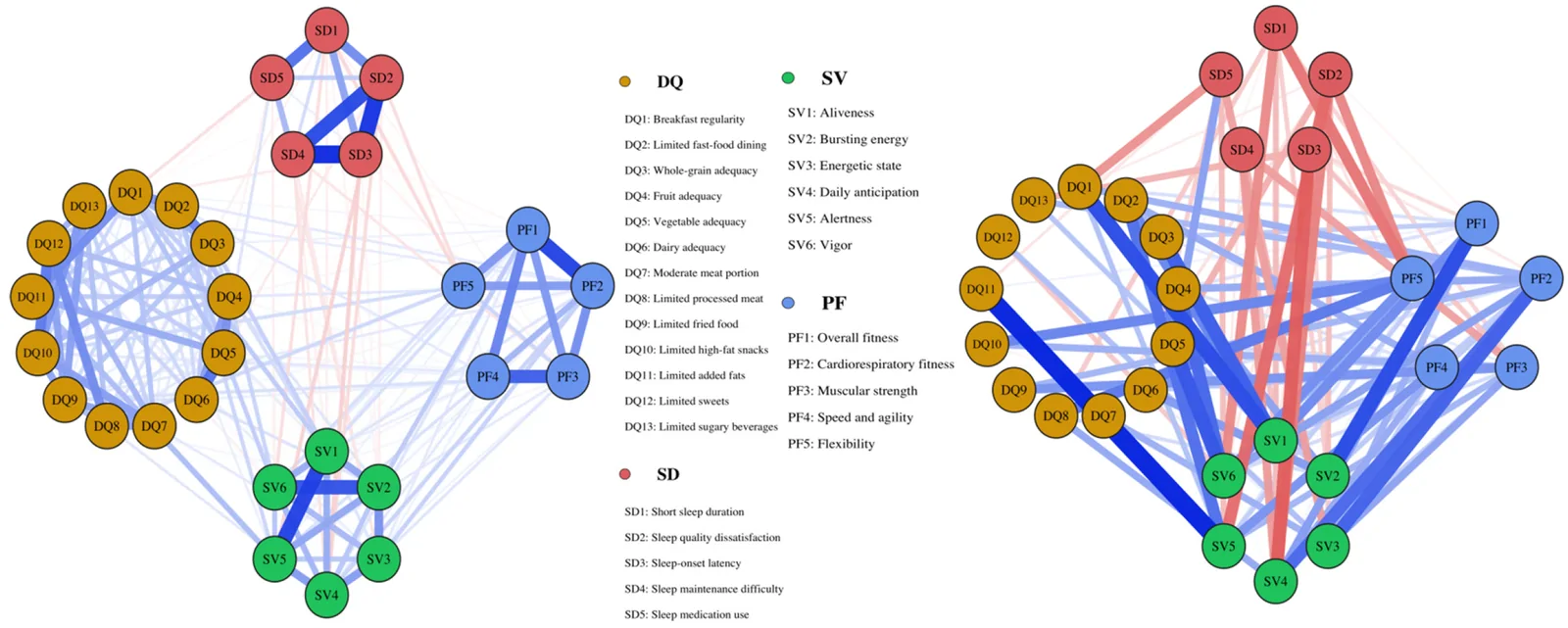
Network structure and bridge network structure of the DQ–SD–SV–PF network.

Figure 3 presents the DQ–SD–SV–PF item-level network and its cross-community connectivity. Within the SD community, relatively strong positive connections were observed among SD2, SD3, and SD4, indicating that these sleep-related problems remained conditionally interdependent after controlling for all other nodes in the network. Within the SV community, SV1, SV2, SV5, and SV6 showed comparatively strong positive connections. The self-rated PF items also formed a coherent positive structure, with PF1–PF5 displaying substantial conditional consistency at the item level.

Clear patterns were also evident across communities. Connections between SD and both SV and PF were predominantly negative, whereas associations between SV and PF were largely positive. Multiple positive cross-community edges were also observed between DQ and both SV and PF, although these were generally weaker than the strongest within-community connections. Taken together, this structure indicates that SV occupied a prominent cross-community position linking dietary behaviors, sleep-related difficulties, and self-rated physical fitness within the broader network of athlete health behaviors and perceived functional status.

**Figure 4:**
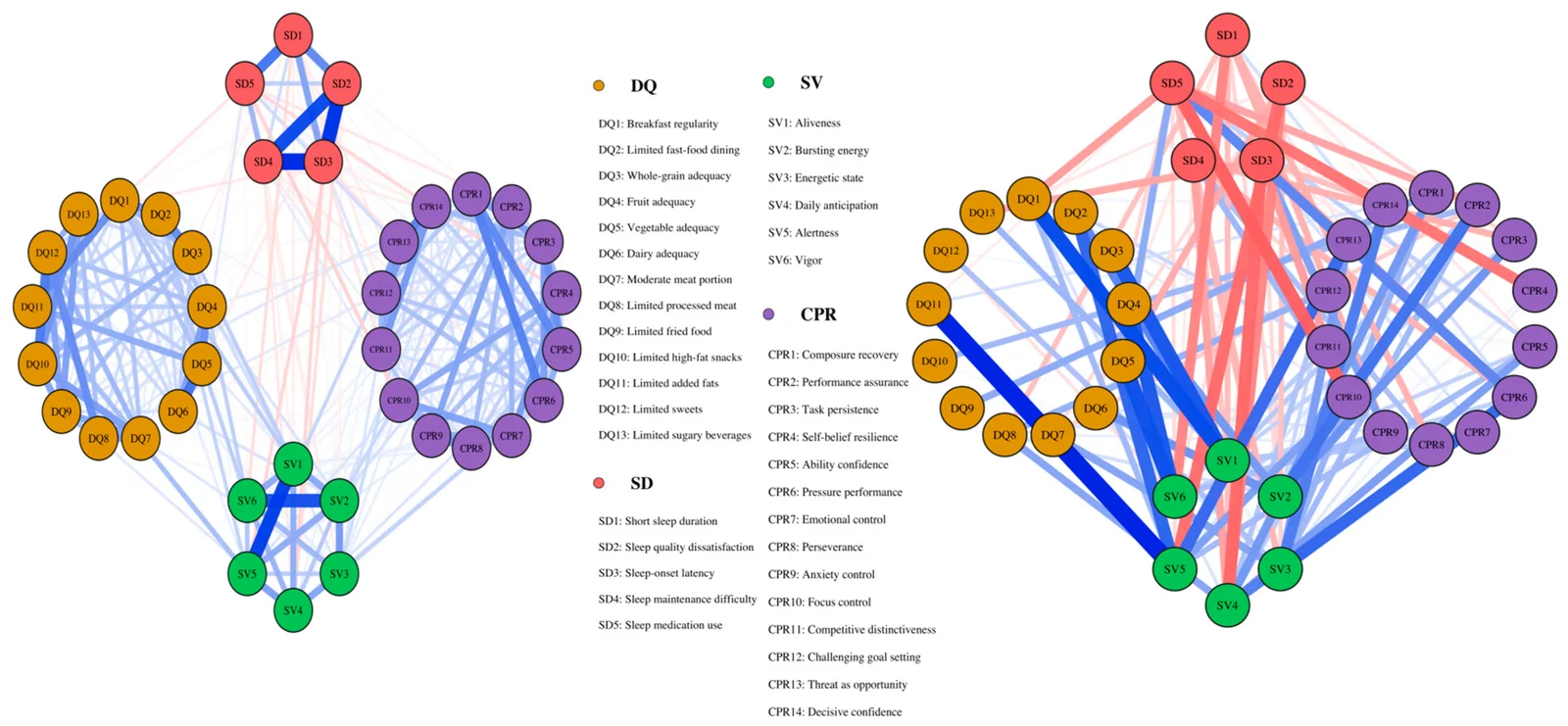
Network structure and bridge network structure of the DQ–SD–SV–CPR network.

Figure 4 presents the DQ–SD–SV–CPR network. The CPR nodes, derived from SMTQ mental-toughness items, formed a relatively cohesive within-community structure, with comparatively strong positive connections among CPR5–CPR8, CPR10, CPR12, and CPR14, indicating that these psychological resources were closely interconnected. At the cross-community level, connections between SD and both SV and CPR were predominantly negative, whereas SV showed positive connections with multiple CPR nodes. DQ also showed cross-community connections with CPR and with nodes in the SD and SV communities. Overall, the relatively concentrated connections between SV and CPR suggest that vitality was prominently linked to the mental-toughness-based CPR measure within the network.

#### 3.5.3. Bridge Centrality

**Figure 5:**
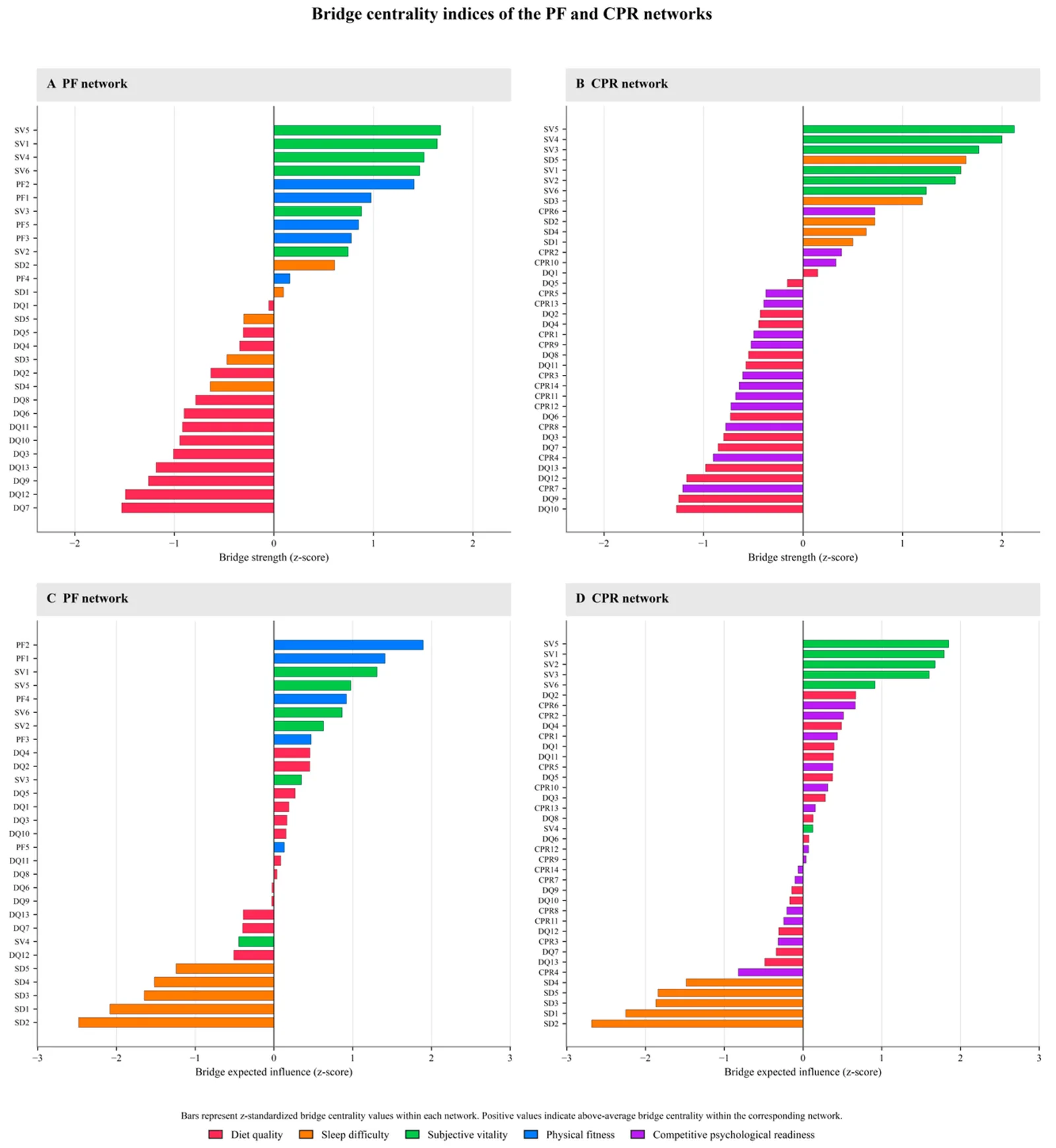
Bridge centrality indices in the PF and CPR networks. Note. Panels (**A**,**B**) present bridge strength in the self-rated physical fitness (PF) and competitive psychological readiness (CPR) networks, respectively; CPR was operationalized using mental toughness. Panels (**C**,**D**) present one-step bridge expected influence in the PF and CPR networks, respectively. Positive values indicate above-average bridge centrality, whereas negative values indicate below-average bridge centrality. Node colors indicate community membership: diet quality, sleep difficulty, subjective vitality, self-rated physical fitness, and competitive psychological readiness.

Figure 5 compares standardized Bridge Strength and one-step Bridge Expected Influence across the PF and CPR networks. In the PF network, SV5, SV1, SV4, and SV6 showed relatively high Bridge Strength, followed by PF2 and PF1, indicating that prominent cross-community connections were concentrated primarily among SV and PF nodes. Bridge Expected Influence further showed that PF2, PF1, SV1, SV5, PF4, SV6, and SV2 had relatively strong net positive cross-community connectivity, whereas SD2, SD1, SD3, and SD4 exhibited more pronounced net negative cross-community connections.

In the CPR network, SV5, SV4, and SV3 showed relatively high Bridge Strength, followed by SD5, SV1, SV2, and SV6. Bridge Expected Influence was relatively high for SV5, SV1, SV2, SV3, and SV6, with CPR6 and CPR2 also showing comparatively high positive values. In contrast, SD2, SD1, SD3, SD5, and SD4 exhibited comparatively pronounced negative Bridge Expected Influence. Overall, the two networks displayed a similar bridging pattern: SV nodes were predominantly characterized by positive cross-community connectivity, whereas SD nodes were primarily characterized by negative cross-community connectivity. It should be emphasized that Bridge Strength reflects the overall magnitude of a node’s connections with other communities, whereas Bridge Expected Influence preserves the direction of edge weights and therefore better characterizes the net positive or negative cross-community connectivity of a node. Because bridge centrality indices were not independently evaluated using case-dropping stability procedures, small differences in their relative rankings should be interpreted cautiously. Moreover, these findings represent conditional associations within a cross-sectional network and should not be interpreted as evidence of temporal ordering or causal relationships.

#### 3.5.4. Network Accuracy and Centrality Stability

Nonparametric bootstrap analyses indicated that the principal network edge-weight estimates showed acceptable accuracy. Case-dropping bootstrap analyses further showed that the CS coefficients for Strength and Expected Influence ranged from 0.672 to 0.750 across the PF and CPR networks, all exceeding 0.50 and supporting the stability of these conventional centrality indices. Bridge centrality measures were used to characterize cross-community connectivity; however, their fine-grained relative rankings were not independently evaluated for stability and should therefore not be interpreted as empirically stable node rankings.

### 3.6. Machine-Learning Prediction Analysis

To further evaluate the relative predictive contributions of the candidate variables to self-rated PF and CPR, separate Ridge regression and XGBoost models were developed for each outcome. Candidate predictors included DQ, SD, SV, sex, age, BMI, daily training duration, weekly training frequency, formal athlete classification, and sport type. Model performance was evaluated using five-fold cross-validation within the training set and a held-out 20% test set.

As shown in Table 5, Ridge regression achieved better holdout performance than the prespecified XGBoost model for both outcomes. For PF, the holdout *R*^2^ values were 0.415 for Ridge regression and 0.378 for XGBoost; for CPR, the corresponding values were 0.341 and 0.327. Thus, under the current predictor set and model specifications, the regularized linear model provided stronger out-of-sample prediction, whereas the nonlinear model offered no additional predictive advantage. This pattern may reflect limited exploitable nonlinear or interaction information, although XGBoost performance may also have been constrained by the prespecified hyperparameters and the sample size relative to model complexity. Because XGBoost was not systematically optimized, these findings should not be interpreted as evidence that nonlinear models are generally ineffective or that the underlying relationships are necessarily linear. Ridge regression was therefore retained as the primary predictive benchmark, with XGBoost and its SHAP-based interpretation serving as complementary analyses of model-specific predictor contributions. The prespecified XGBoost hyperparameters, selected Ridge penalty parameters, and Ridge coefficients are reported in Supplementary Tables S1–S3, respectively, and the complete SHAP results for the PF and CPR models in Supplementary Tables S4 and S5. The same relative pattern emerged across 100 repeated 80/20 splits: Ridge yielded higher mean test-set *R*^2^ than XGBoost for PF (0.412 ± 0.038 vs. 0.383 ± 0.038) and CPR (0.340 ± 0.040 vs. 0.330 ± 0.042), with corresponding RMSE and MAE results (Supplementary Table S6). The prespecified holdout estimates were close to the repeated-split means, suggesting limited sensitivity to sample allocation; however, all predictive evaluations remained internal to the same cohort.

**Figure 6:**
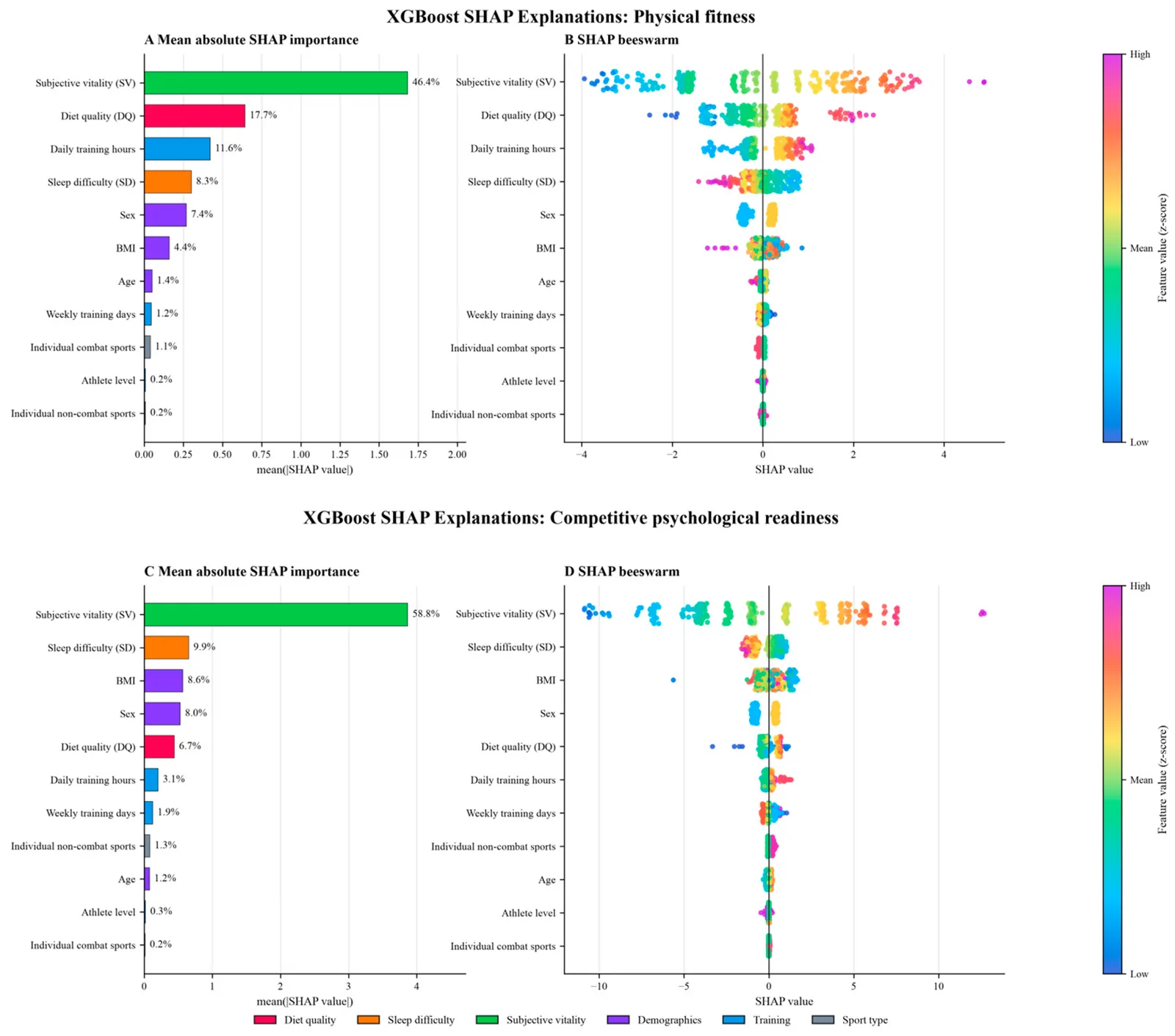
SHAP-based interpretation of the XGBoost models predicting PF and CPR. Note. XGBoost used prespecified hyperparameters and did not outperform Ridge regression in holdout prediction. Therefore, SHAP values are presented as a supplementary interpretation of the prespecified nonlinear model rather than as the primary explanation of the best-performing predictive model. SHAP importance reflects model-specific predictive contribution and does not represent a causal effect.

Figure 6 presents the SHAP-based interpretation of the XGBoost models for PF and CPR. In the PF model, SV had the highest mean absolute SHAP importance, accounting for 46.4% of the total relative importance, followed by DQ (17.7%), daily training duration (11.6%), SD (8.3%), sex (7.4%), and BMI (4.4%). In the CPR model, SV again showed the highest relative importance (58.8%), followed by SD (9.9%), BMI (8.6%), sex (8.0%), DQ (6.7%), and daily training duration (3.1%).

The machine-learning results showed a pattern broadly consistent with the preceding analyses within the same sample. SV had the highest relative SHAP importance in both XGBoost models, whereas DQ ranked more prominently in the PF model than in the CPR model. However, because all analyses were conducted using the same cross-sectional self-report dataset, this convergence should not be considered independent validation. Moreover, the reported SHAP percentages represent relative predictive contributions within a given model; they do not correspond to explained variance, cannot be used to compare effect magnitudes across the two outcomes, and should not be interpreted causally.

## 4. Discussion

Overall, the observed association patterns were broadly consistent with the prespecified association hypotheses. Higher DQ was significantly associated with both higher self-rated PF and higher CPR among collegiate athletes, with CPR operationalized using mental toughness. The models also yielded statistically significant cross-sectional indirect-association estimates involving SD alone, SV alone, and SD and SV in the prespecified order. These estimates represent statistical decompositions of associations measured at a single time point and should not be interpreted as evidence of temporal or causal mechanisms. Network analysis further indicated that SD-related nodes were predominantly characterized by negative cross-community connectivity, whereas SV-related nodes showed comparatively prominent positive connections across construct communities. In the complementary XGBoost models fitted using prespecified hyperparameters, SV showed the highest relative SHAP importance for both PF and CPR. Taken together, these findings provide a conceptual basis for examining DQ, sleep-related recovery, and SV alongside training within an integrated framework for CF. Whether interventions targeting these factors can improve self-rated PF or CPR remains to be tested in longitudinal and experimental research.

### 4.1. Diet Quality in Relation to Self-Rated Physical Fitness and Competitive Psychological Readiness

The results showed that DQ was significantly and positively associated with both self-rated PF and CPR, supporting H1a and H1b. Consistent with previous research, better DQ has been associated with superior physical fitness and task-performance outcomes [62]. From a physical-functioning perspective, higher DQ may reflect dietary patterns that are more conducive to recovery and training adaptation. The established sport-nutrition literature indicates that adequate energy and macronutrient intake can support glycogen restoration, muscle protein synthesis, tissue repair, and immune function, whereas low energy availability is associated with physiological disruption and impaired performance [12,33]. These established mechanisms provide general physiological context for the observed association; however, because the REAP-S did not quantify energy or nutrient intake, determine energy availability, or assess glycogen restoration, muscle protein synthesis, or other recovery-related processes, they should not be interpreted as mechanisms demonstrated by the present data. Within the athlete health–performance framework, nutrition should therefore not be viewed as an ancillary factor external to performance, but rather as a fundamental resource for maintaining physical functional capacity and supporting training adaptation [1]. Accordingly, future intervention studies could examine whether nutritional management extending beyond daily energy provision supports recovery, training adaptation, and perceived physical functioning in collegiate athletes.

Notably, the adjusted association of DQ with CPR was positive but small (*β* = 0.048, 95% CI [0.003, 0.093]) and was numerically weaker than the corresponding association with self-rated PF (*β* = 0.208, 95% CI [0.166, 0.250]). Its statistical significance should therefore not be interpreted as evidence of a substantial or practically important association. This difference may reflect variation in the proximity of DQ to different dimensions of CF. Meta-analytic evidence indicates that low energy availability is associated with poorer training responses and reductions in endurance, power, agility, coordination, and related performance capacities [63], providing broader physiological context for the comparatively stronger association observed with self-rated PF. Because neither energy availability nor objective physical performance was measured in the present study, this evidence should not be interpreted as identifying the mechanism underlying the observed DQ–PF association. By comparison, CPR was operationalized using mental toughness, a psychological regulatory resource that may depend more strongly on motivational processes and the satisfaction of basic psychological needs. Previous research has shown that satisfaction of basic psychological needs is associated with greater mental toughness and better athletic performance, supporting the importance of motivational and self-regulatory processes in the development of psychological resilience in sport [64]. This distinction may be particularly relevant for collegiate athletes. As a typical dual-career population, they must simultaneously manage academic responsibilities, training demands, and competitive commitments, while their psychological readiness for competition may also be shaped by time pressure, competitive stress, and the allocation of limited personal resources [9]. Accordingly, the comparatively weaker association between DQ and CPR may suggest that this psychological dimension of CF is shaped by a broader combination of recovery status, resource allocation, and contextual stressors than is self-rated PF.

The network findings further refined the observed differences between DQ and the two dimensions of CF. In the PF network, PF1 and PF2 emerged as relatively prominent nodes with positive cross-community connectivity. This pattern may reflect the fact that overall perceived fitness integrates athletes’ evaluations across multiple physical domains, whereas perceived cardiorespiratory fitness may capture their appraisal of endurance-related capacity, training adaptation, and recovery status. PF1 and PF2 were therefore among the PF items showing relatively prominent connections with variables outside their own community. By contrast, cross-community connectivity in the CPR network was more strongly concentrated around SV. Within the CPR community, CPR6 and CPR2 also showed relatively prominent positive bridging patterns, placing performance under pressure and performance assurance among the mental-toughness facets more closely connected with experiences outside the CPR community. Conceptually, both facets involve maintaining confidence and effective self-regulation when energetic resources are challenged, which may help explain their connections with SV within broader recovery and self-regulation frameworks [2,64]. These patterns offer theoretically informed hypotheses for replication rather than evidence of established mechanisms. This interpretation was broadly consistent with the predictive analyses: DQ ranked more highly in relative predictive importance for PF than for CPR, whereas SV showed the highest relative SHAP importance within the CPR XGBoost model. Taken together, the network analysis identified specific cross-construct connections that were not visible in the scale-level models, while the machine-learning analysis provided complementary information regarding their predictive relevance. For collegiate athletes managing simultaneous academic and competitive demands, high-quality dietary behavior may therefore be considered a relevant correlate within the broader CF framework. Its association was more pronounced for perceived physical functioning, whereas competitive psychological functioning appeared to covary with a broader combination of nutritional, recovery-related, and psychological resources.

### 4.2. Sleep-Difficulty Mediation of the Diet Quality–Competitive Functioning Association

Higher DQ was associated with lower SD, and the models yielded statistically significant indirect-association estimates involving SD for both self-rated PF and CPR. These patterns were consistent with H2a and H2b. This pattern is consistent with previous research on sleep and recovery in athletes [65]. Related reviews have further suggested that athletes’ sleep may be associated with dietary composition, nutrient timing, and the consumption of stimulants such as caffeine, while the relationship between sleep and nutrition may also be bidirectional [66]. Sleep is also essential for physiological and cognitive recovery following training, and impaired sleep may be accompanied by insufficient recovery, increased fatigue, and subsequent deterioration in athletic functioning [67,68]. The present findings indicate that DQ, SD, and the two outcomes covaried in the theoretically specified models. They do not establish that DQ preceded changes in sleep or that sleep changes subsequently altered either outcome.

SD also showed a significant indirect association between DQ and CPR, although the corresponding path coefficients were numerically smaller than those observed for PF. From a recovery–adaptation perspective, the effects of training depend on an appropriate balance between training stimuli and subsequent recovery, with inadequate recovery often becoming evident first through persistent fatigue, impaired adaptation, and reduced physical functioning [69]. By contrast, CPR was operationalized in the present study using mental toughness. Mental toughness may exhibit a degree of cross-situational stability while also remaining responsive to training experience and psychological skills development, and it represents a broader psychological resource that supports sustained goal pursuit and coping with competitive pressure [70,71]. Consequently, even when experiencing SD, athletes may retain a certain level of psychological readiness through previously developed psychological skills, competitive experience, and continued goal engagement. Among collegiate athletes, academic schedules, training times, and competition demands may collectively restrict opportunities for recovery, making insufficient sleep more likely to manifest initially as physical fatigue. Competitive psychological functioning, however, may additionally be shaped by contextual influences such as academic stress, role conflict, and coach support [72,73]. SD may therefore constitute an important recovery-related context for CPR without being sufficient, on its own, to explain individual differences in psychological readiness.

The network and machine-learning findings further qualified this interpretation. Across both the PF and CPR networks, SD2, SD1, SD3, and SD4 showed relatively pronounced negative Bridge Expected Influence, indicating that the associations of SD with PF and CPR were not concentrated in a single sleep-related problem. Instead, they involved multiple aspects of sleep, including adequacy, continuity, and perceived recovery quality. SD2 may capture athletes’ overall perception of nocturnal recovery quality, SD1 directly reflects reduced time available for recovery, and SD3 and SD4 represent difficulties with sleep initiation and sleep maintenance, respectively. The machine-learning analyses further showed that SD accounted for 8.3% and 9.9% of relative SHAP importance within the PF and CPR XGBoost models, respectively, indicating that SD retained non-negligible predictive information for both outcomes. These SHAP percentages, however, represent relative predictive contributions within each individual model and should not be interpreted as evidence that SD exerts a stronger influence on psychological functioning than on physical functioning. The network analysis therefore indicated that sleep-related cross-community connectivity was distributed across several aspects of sleep rather than concentrated in a single item, while the XGBoost models showed that SD retained relative predictive information after the other predictors had been considered. These patterns provide a rationale for future intervention studies among collegiate athletes managing concurrent academic and competitive demands, to evaluate sleep duration, sleep quality, sleep-onset efficiency, and sleep continuity alongside training schedules, academic commitments, and nutritional planning.

### 4.3. Subjective-Vitality Mediation of the Diet Quality–Competitive Functioning Association

The models yielded statistically significant cross-sectional indirect-association estimates involving SV for both PF and CPR, consistent with H3a and H3b. These estimates were larger than those involving SD alone; however, this difference reflects model-based magnitude rather than temporal proximity or causal importance. One possible interpretation is that SV captures athletes’ perceived availability of physical and psychological energy, which may be closely related to both dietary behavior and competitive functioning. Among university students, healthier dietary patterns have been associated with higher SV, suggesting that habitual dietary behavior covaries with subjective energy experience and psychological functioning [34]. In sport settings, SV has also been linked to psychological resources and goal-regulation processes. Ommundsen et al. found that psychological need satisfaction and autonomous motivation significantly predicted SV among adolescent soccer players [74], while longitudinal research in collegiate athletes linked changes in goal motivation with changes in SV [75]. Taken together, this evidence suggests that SV is not simply a reflection of DQ but a broader subjective functional state associated with dietary behavior, the competitive environment, and psychological resources.

Notably, the association between SV and CPR was numerically stronger than that between SV and self-rated PF. One possible explanation is that SV inherently encompasses psychological experiences such as alertness, energy, and active engagement, which are functionally closer to the confidence maintenance and stress-coping processes represented by mental toughness. Previous research among high-level combat-sport athletes has similarly linked SV with mental toughness and psychological well-being [76]. Although that population differs from the present collegiate sample in competitive level, the conceptual convergence supports examining whether this association is robust across sporting contexts rather than assuming direct generalizability. Other work has also suggested that evaluations of performance around the onset of competition may incorporate state-related cues reflecting both psychological and physical vitality [77]. By contrast, athletes’ ratings of PF may also incorporate their perceptions of training load, sport-specific capacity, body composition, and technical proficiency; SV is therefore likely to account for only part of the variation in perceived physical functioning. SV may therefore represent a salient psychological resource associated with everyday health behaviors and competitive psychological functioning, while also informing athletes’ evaluations of physical functioning. These observations provide a rationale for future studies to test whether athlete-support programs benefit from considering current energy, alertness, and sustained engagement alongside objective training and sleep indicators.

Across the PF and CPR networks, SV5, SV1, SV6, and related energy-experience nodes showed relatively high Bridge Strength and positive Bridge Expected Influence, indicating that the cross-community connectivity of SV was distributed across multiple interrelated vitality experiences rather than being concentrated in a single item. The exploratory XGBoost analyses showed a broadly similar pattern within the same sample, with SV exhibiting the highest relative SHAP importance in both prespecified models. This overlap provides complementary rather than independent information: the network analysis identified vitality experiences with relatively prominent cross-community connections, whereas SHAP characterized the model-specific predictive contribution of overall SV. These findings identify SV as a relevant construct for further investigation within the broader assessment of CF, but they do not establish it as the most important determinant or a causal intervention target.

### 4.4. Serial Indirect Association Through Sleep Difficulty and Subjective Vitality

Indirect-association analysis indicated an ordered pattern among DQ, SD, SV, PF, and CPR spanning recovery status and perceived resource availability. Within the proposed model, higher DQ was associated with lower SD, lower SD with higher SV, and higher SV with higher self-rated PF and CPR scores, consistent with H4a and H4b. Notably, in both outcome models, the ordered indirect-association estimate involving both SD and SV was significantly larger than the estimate involving SD alone but significantly smaller than that involving SV alone. This asymmetric pattern suggests that SD is related not only to disrupted recovery but also to perceived physical and psychological resource availability, whereas SV showed stronger associations with the measured dimensions of CF. DQ also remained significantly associated with SV after SD was included in the model, indicating that the DQ–SV association was not fully accounted for by SD. Taken together, these patterns are consistent with the proposed recovery–resource ordering.

This pattern is theoretically consistent with a recovery–resource perspective. SD reflects disruption to nocturnal recovery, whereas SV captures the physical and psychological energy that athletes perceive as available and mobilizable during waking hours. Conservation of resources theory proposes that restorative processes help replenish resources depleted by everyday demands and training, whereas inadequate recovery may restrict the resources available for subsequent functioning [38]. Similarly, the recovery–performance framework emphasizes the importance of maintaining an appropriate balance between training load and recovery for optimal functional status [2]. From this perspective, DQ may be understood as a modifiable health-related condition, SD as an indicator of recovery-related disruption, SV as a proximal resource state, and PF and CPR as the measured physical and psychological dimensions of CF. The significant ordered indirect associations observed in both outcome models are consistent with a broader “health behavior–recovery status–resource experience” framework for understanding how DQ relates to different dimensions of CF.

Because the present study used a cross-sectional design, these findings should not be interpreted as evidence that better DQ sequentially improves sleep, increases SV, and ultimately enhances CF, nor do they establish that SD temporally precedes SV. Rather, the observed associations were consistent with the proposed sequence, while alternative or reciprocal relationships remain possible. The assessment context further qualifies this interpretation. Competition check-in provided a common, performance-salient measurement window, but reports of SD, SV, self-rated PF, and CPR collected after arrival may also have reflected recent travel, tapering, acute fatigue, competition anticipation, or pre-competition stress. Unmeasured training, injury, nutritional, and psychological factors may likewise have contributed to the observed pattern. The covariation may therefore combine relatively stable between-athlete differences with temporary responses to the immediate competitive environment. For athletes simultaneously managing academic, training, and competition demands, the observed pattern provides a rationale for future intervention research integrating dietary behavior, sleep-related recovery, daytime vitality, training-load management, and psychological skills development. Whether such an integrated approach improves self-rated PF or CPR requires longitudinal and experimental testing.

## 5. Limitations

Several limitations should be acknowledged. First, the cross-sectional design precludes establishing temporal precedence or causal relationships among the modeled associations. Although SD was positioned before SV on theoretical grounds, this ordering cannot be distinguished from alternative or reciprocal associations. Future studies should therefore employ multiwave longitudinal designs, intensive diary methods, or experimental approaches to clarify the temporal structure of these relationships.

Second, all core variables were assessed using self-report instruments at a single measurement occasion and may therefore be susceptible to recall bias, social desirability effects, and shared method variance. Although the ULMC results indicated that the method factor accounted for only a small proportion of variance, this analysis cannot eliminate biases arising from same-source self-report measurement. In addition, the REAP-S is a brief dietary-behavior screener and does not quantify dietary intake, nutrient adequacy, energy availability, or physiological recovery; the related physiological mechanisms should therefore be regarded as literature-based context rather than processes demonstrated by the present data. Future research could combine dietary records or 24-h dietary recalls with energy-expenditure assessment, actigraphy, objective fitness testing, and competition-performance data.

Third, PF represented self-rated physical fitness, whereas CPR was operationalized using mental toughness to capture the psychological dimension of CF. CF was therefore treated as an organizing framework rather than as a separately measured composite construct. These indicators should not be considered equivalent to objective athletic performance or to the full construct of pre-competition psychological readiness. Future studies should incorporate objective fitness assessments, competition data, coach evaluations, and repeated pre-competition measurements to strengthen the content validity of CF assessment.

Fourth, although the statistical models adjusted for major demographic and habitual training characteristics, residual confounding may remain. Current injury status, previous training history, recent training load, competition phase, recent weight-loss practices, energy availability, nutritional supplementation, psychological stress, and travel were not fully accounted for. Several of these factors may be associated simultaneously with dietary behavior, sleep, vitality, and self-rated functioning. Moreover, assessment during competition check-in may have introduced shared state-related variation associated with travel, tapering, acute fatigue, competition anticipation, or pre-competition stress. Future studies should prespecify a more comprehensive set of covariates and conduct repeated assessments across training and competition phases to distinguish contextual fluctuations from more stable associations.

Fifth, convenience sampling from provincial-level university sporting events in Yunnan and Guizhou limits the broader applicability of the findings. Participants were university students engaged in systematic sport-specific training who represented their institutions in competition, although 76.42% did not hold a formal national athlete classification. The sample also combined team, individual combat, and individual non-combat sports in markedly unequal proportions. Although broad sport category was included as a covariate, this adjustment may not capture discipline-specific differences in dietary practices, weight-management demands, training load, sleep patterns, or competition structure. The findings are therefore most directly applicable to collegiate athletes competing within provincial university sport systems and should be extrapolated cautiously across regions, institutions, sport disciplines, and competitive levels. Future studies should recruit more geographically diverse samples with balanced representation across sport categories and prospectively test whether these association patterns vary by sport discipline and competitive level.

Sixth, the cross-sectional networks reflect conditional associations among items rather than causal effects, while SHAP importance represents only the relative predictive contribution of variables within a given model. Neither approach can establish causality. Because XGBoost used prespecified rather than systematically optimized hyperparameters, its SHAP results should be regarded as exploratory and model-dependent. Although the repeated 80/20 analyses reduced dependence on a single sample allocation, all predictive evaluations remained internal to the same cohort and therefore do not establish transportability to other athlete populations or competitive settings. Similarities between the network and predictive findings cannot constitute independent validation because both analyses used the same participants and dataset. In addition, the stability of bridge-centrality rankings was not independently evaluated using case-dropping procedures; consequently, small differences in the relative rankings of bridge nodes should be interpreted cautiously, and the item-level bridge patterns should be regarded as descriptive and hypothesis-generating rather than as evidence of established mechanisms or intervention targets. Future research should employ longitudinal network models, replicate network estimation in independent samples, and conduct external validation of machine-learning models to assess the stability and generalizability of both structural and predictive findings.

## 6. Conclusions

Higher DQ was associated with higher self-rated PF and CPR scores in this sample of collegiate athletes competing at provincial-level university events, with CPR operationalized using mental toughness. Significant cross-sectional indirect associations were observed involving SD alone, SV alone, and SD and SV in the prespecified order. The network and machine-learning analyses provided complementary exploratory information about structural and predictive patterns involving SV within this dataset. Overall, the findings provide a conceptual basis for considering dietary behavior, sleep-related recovery, and daytime vitality within an integrated framework for understanding CF in collegiate athletes. Whether modifying these factors improves self-rated PF or CPR requires longitudinal and experimental testing.

## Figures and Tables

**Table 1:** Participant characteristics and study variables by sex.

Characteristics	Total (*n* = 1641)	Male (*n* = 1100)	Female (*n* = 541)	*p*	Effect Size
Age, *M* ± *SD*	20.53 ± 1.46	20.50 ± 1.47	20.59 ± 1.43	0.258	−0.06
Height, cm	173.86 ± 8.75	178.22 ± 6.37	165.01 ± 5.73	<0.001	2.14
Weight, kg	67.83 ± 9.87	71.32 ± 8.86	60.74 ± 7.78	<0.001	1.24
BMI	22.38 ± 2.37	22.44 ± 2.35	22.28 ± 2.42	0.223	0.06
Daily training hours	1.99 ± 0.90	1.99 ± 0.89	1.98 ± 0.91	0.874	0.01
Weekly training days	4.50 ± 0.93	4.49 ± 0.95	4.51 ± 0.90	0.703	−0.02
DQ	29.89 ± 3.71	29.61 ± 3.65	30.47 ± 3.79	<0.001	−0.23
SD	5.33 ± 3.34	5.06 ± 3.26	5.90 ± 3.43	<0.001	−0.25
SV	3.98 ± 1.01	4.03 ± 1.02	3.90 ± 1.00	0.014	0.13
PF	14.77 ± 3.96	15.10 ± 3.93	14.11 ± 3.93	<0.001	0.25
CPR	35.43 ± 8.44	36.09 ± 8.30	34.09 ± 8.57	<0.001	0.24
Grade, *n* (%)				0.850	0.03
Freshman	362 (22.06)	251 (22.82)	111 (20.52)		
Sophomore	364 (22.18)	239 (21.73)	125 (23.11)		
Junior	434 (26.45)	290 (26.36)	144 (26.62)		
Senior	304 (18.53)	204 (18.55)	100 (18.48)		
Graduate student	177 (10.79)	116 (10.55)	61 (11.28)		
formal athlete classification				0.231	0.05
No formal classification	1254 (76.42)	843 (76.64)	411 (75.97)		
National second-class	292 (17.79)	186 (16.91)	106 (19.59)		
National first-class	75 (4.57)	57 (5.18)	18 (3.33)		
Master-level or above	20 (1.22)	14 (1.27)	6 (1.11)		
Sport type				0.392	0.03
Team sports	990 (60.33)	663 (60.27)	327 (60.44)		
Individual combat sports	380 (23.16)	247 (22.45)	133 (24.58)		
Individual non-combat sports	271 (16.51)	190 (17.27)	81 (14.97)		
Diet quality category				<0.001	0.09
Below reference (<32)	1092 (66.54)	765 (69.55)	327 (60.44)		
At/above reference (≥32)	549 (33.46)	335 (30.45)	214 (39.56)		
Sleep difficulty category				<0.001	0.11
None (0–4)	721 (43.94)	516 (46.91)	205 (37.89)		
Mild (5–7)	507 (30.90)	342 (31.09)	165 (30.50)		
Moderate (8–10)	274 (16.70)	162 (14.73)	112 (20.70)		
Severe (11–17)	139 (8.47)	80 (7.27)	59 (10.91)		

Note. BMI = body mass index; DQ = diet quality; SD = sleep difficulty; SV = subjective vitality; PF = self-rated physical fitness; CPR = competitive psychological readiness operationalized using mental toughness. Data are presented as *M* ± *SD* or n (%). The REAP-S reference value of 32 was used descriptively; DQ remained continuous in all primary analyses. Sex comparisons used Welch’s *t* tests and Hedges’ *g* for continuous variables and Pearson’s *χ*^2^ tests and Cramér’s *V* for categorical variables. Hedges’ *g* was calculated as male minus female (positive = higher in men; negative = higher in women).

**Table 2:** Correlations among the main study variables.

Variable	1	2	3	4	5
1. DQ	1				
2. SD	−0.282 ***	1			
3. SV	0.453 ***	−0.479 ***	1		
4. PF	0.428 ***	−0.382 ***	0.597 ***	1	
5. CPR	0.295 ***	−0.340 ***	0.580 ***	0.556 ***	1

Note. DQ = diet quality; SD = sleep difficulty; SV = subjective vitality; PF = self-rated physical fitness; CPR = competitive psychological readiness operationalized using mental toughness. *** *p* < 0.001.

**Table 3:** Path coefficients from the cross-sectional models.

Model	Outcome	Predictor	*β*	*SE*	*t*	95% CI
Intermediate-variable equation	SD	DQ	−0.299 ***	0.024	−12.663	[−0.345, −0.253]
Intermediate-variable equation	SV	DQ	0.354 ***	0.021	16.722	[0.313, 0.396]
Intermediate-variable equation	SV	SD	−0.370 ***	0.021	−17.479	[−0.412, −0.329]
Model 1	PF	DQ	0.208 ***	0.022	9.651	[0.166, 0.250]
Model 1	PF	SD	−0.101 ***	0.022	−4.635	[−0.143, −0.058]
Model 1	PF	SV	0.449 ***	0.023	19.284	[0.403, 0.494]
Model 2	CPR	DQ	0.048 *	0.023	2.081	[0.003, 0.093]
Model 2	CPR	SD	−0.069 **	0.023	−2.997	[−0.114, −0.024]
Model 2	CPR	SV	0.521 ***	0.025	21.091	[0.473, 0.570]

Note. DQ = diet quality; SD = sleep difficulty; SV = subjective vitality; PF = self-rated physical fitness; CPR = competitive psychological readiness operationalized using mental toughness. Model 1 predicted PF, and Model 2 predicted CPR. All models controlled for sex, age, BMI, formal athlete classification, daily training hours, weekly training days, and sport type. Sport type was dummy-coded, with team sports as the reference group. *β* = standardized regression coefficient. * *p* < 0.05, ** *p* < 0.01, *** *p* < 0.001.

**Table 4:** Bootstrap estimates of the indirect effects.

Model	Component	Model Term	Estimate	BootSE	95% Bootstrap CI
Model 1: PF	Total	Total Indirect Effect	0.239	0.014	[0.212, 0.267]
	Ind1	DQ → SD → PF	0.030	0.007	[0.018, 0.044]
	Ind2	DQ → SV → PF	0.159	0.012	[0.135, 0.184]
	Ind3	DQ → SD → SV → PF	0.050	0.005	[0.040, 0.061]
	C1	Ind1 − Ind2	−0.129	0.016	[−0.159, −0.098]
	C2	Ind1 − Ind3	−0.020	0.008	[−0.036, −0.004]
	C3	Ind2 − Ind3	0.109	0.013	[0.084, 0.135]
Model 2: CPR	Total	Total Indirect Effect	0.263	0.015	[0.233, 0.293]
	Ind1	DQ → SD → CPR	0.021	0.007	[0.007, 0.035]
	Ind2	DQ → SV → CPR	0.185	0.014	[0.159, 0.212]
	Ind3	DQ → SD → SV → CPR	0.058	0.006	[0.046, 0.070]
	C1	Ind1 − Ind2	−0.164	0.017	[−0.197, −0.132]
	C2	Ind1 − Ind3	−0.037	0.009	[−0.057, −0.020]
	C3	Ind2 − Ind3	0.127	0.014	[0.099, 0.156]

Note. DQ = diet quality; SD = sleep difficulty; SV = subjective vitality; PF = self-rated physical fitness; CPR = competitive psychological readiness operationalized using mental toughness. Model 1 predicted PF, and Model 2 predicted CPR. Bootstrap CIs were based on 5000 resamples. Ind1 = DQ → SD → outcome; Ind2 = DQ → SV → outcome; Ind3 = DQ → SD → SV → outcome. Arrows indicate the prespecified model order, not temporal precedence or causality.

**Table 5:** Predictive performance of Ridge regression and prespecified XGBoost models.

Outcome	Model	Training Set	Holdout Set	5-Fold CV RMSE	5-Fold CV MAE	5-Fold CV *R*^2^	Holdout RMSE	Holdout MAE	Holdout *R*^2^
PF	Ridge	1312	329	3.032 ± 0.164	2.475 ± 0.161	0.410 ± 0.062	3.006	2.429	0.415
PF	XGBoost	1312	329	3.109 ± 0.179	2.512 ± 0.159	0.379 ± 0.074	3.100	2.502	0.378
CPR	Ridge	1312	329	6.780 ± 0.207	5.525 ± 0.146	0.338 ± 0.051	7.096	5.774	0.341
CPR	XGBoost	1312	329	6.878 ± 0.248	5.600 ± 0.156	0.318 ± 0.054	7.172	5.887	0.327

Note. The prespecified 80/20 split used random seed 20260716. Five-fold cross-validation was conducted within the training set, and both models were evaluated on the same holdout set. XGBoost used prespecified hyperparameters without holdout-based tuning and was treated as an exploratory nonlinear comparator.

## Data Availability

The datasets generated and/or analyzed during the current study are available from the corresponding author upon reasonable request due to participant privacy protection requirements specified in the ethical approval protocol.

## Supplementary Materials

The following supporting information can be downloaded at: https://www.mdpi.com/article/10.3390/nu18203385/s1, Table S1: Prespecified XGBoost hyperparameters; Table S2: Ridge regression penalty parameters; Table S3: Ridge regression coefficients; Table S4: Complete SHAP feature importance for the self-rated physical fitness model; Table S5: Complete SHAP feature importance for the competitive psychological readiness model; Table S6: Predictive performance across 100 repeated 80/20 train/test splits.

## Abbreviations

The following abbreviations are used in this manuscript:

CF	Competitive functioning, used as an organizing conceptual framework
PF	Self-perceived physical fitness
CPR	Competitive psychological readiness, operationalized using mental toughness
DQ	Diet quality
SD	Sleep difficulty
SV	Subjective vitality
